# Anti-Inflammatory Effects of Chloranthalactone B in LPS-Stimulated RAW264.7 Cells

**DOI:** 10.3390/ijms17111938

**Published:** 2016-11-22

**Authors:** Xueqin Li, Jun Shen, Yunyao Jiang, Ting Shen, Long You, Xiaobo Sun, Xudong Xu, Weicheng Hu, Haifeng Wu, Gongcheng Wang

**Affiliations:** 1 Department of Gerontology, Huai’an First People’s Hospital, Nanjing Medical University, Huaian 223300, China; doctorlixq@sina.com; 2 Department of Neurology, Huai’an Hospital Affiliated of Xuzhou Medical College and Huai’an Second People’s Hospital, Huaian 223300, China; sj30918@163.com; 3 Xiyuan Hospital, China Academy of Chinese Medical Sciences, Beijing 100091, China; yunyao86@126.com; 4 Shineway Pharmaceutical Group Co., Ltd., Shijiazhuang 051430, China; 5 Jiangsu Collaborative Innovation Center of Regional Modern Agriculture & Environmental Protection, Huaiyin Normal University, Huaian 223300, China; shenting1019@163.com (T.S.); Long_YOU66@163.com (L.Y.); 6 Key Laboratory of Bioactive Substances and Resources Utilization of Chinese Herbal Medicine, Ministry of Education, Institute of Medicinal Plant Development, Peking Union Medical College and Chinese Academy of Medical Sciences, Beijing 100193, China; xbsun@implad.ac.cn (X.S.); xdxu@implad.ac.cn (X.X.); 7 Department of Urology, Huai’an First People’s Hospital, Nanjing Medical University, 6 Beijing West Road, Huaian 223300, China; 8 Jiangsu Key Laboratory for Eco-Agricultural Biotechnology around Hongze Lake, Huaiyin Normal University, Huaian 223300, China

**Keywords:** *Sarcandra glabra*, sesquiterpene, chloranthalactone B, inflammation

## Abstract

Chloranthalactone B (CTB), a lindenane-type sesquiterpenoid, was obtained from the Chinese medicinal herb *Sarcandra glabra*, which is frequently used as a remedy for inflammatory diseases. However, the anti-inflammatory mechanisms of CTB have not been fully elucidated. In this study, we investigated the molecular mechanisms underlying these effects in lipopolysaccharide (LPS)-stimulated RAW264.7 macrophages. CTB strongly inhibited the production of nitric oxide and pro-inflammatory mediators such as prostaglandin E_2_, tumor necrosis factor α (TNF-α), interleukin-1β (IL-1β), and IL-6 in RAW264.7 cells stimulated with LPS. A reverse-transcription polymerase chain reaction assay and Western blot further confirmed that CTB inhibited the expression of inducible nitric oxide synthase, cyclooxygenase-2, TNF-α, and IL-1β at the transcriptional level, and decreased the luciferase activities of activator protein (AP)-1 reporter promoters. These data suggest that inhibition occurred at the transcriptional level. In addition, CTB blocked the activation of p38 mitogen-activated protein kinase (MAPK) but not c-Jun N-terminal kinase or extracellular signal-regulated kinase 1/2. Furthermore, CTB suppressed the phosphorylation of MKK3/6 by targeting the binding sites via formation of hydrogen bonds. Our findings clearly show that CTB inhibits the production of inflammatory mediators by inhibiting the AP-1 and p38 MAPK pathways. Therefore, CTB could potentially be used as an anti-inflammatory agent.

## 1. Introduction

Inflammation is an important part of the complex physiological defense process that is triggered by infection, toxin exposure, tissue injury, or exposure to endotoxins such as lipopolysaccharide (LPS) [1,2]. It is a necessary process that involves the innate and adaptive immune systems, resulting in the production of pro-inflammatory cytokines [3]. However, uncontrolled or prolonged inflammatory responses are harmful and are involved in the pathogenesis of many diseases, including cancer, diabetes, arthritis, Alzheimer’s disease, cardiovascular disease, atherosclerosis, multiple sclerosis, and neurodegenerative diseases [4,5]. Macrophages, a major inflammatory and immune effector cell type, play crucial roles in the immune response, and produce both pro-inflammatory cytokines and inflammatory mediators [6]. LPS, a well-known macrophage activator, is recognized by pattern-recognition receptors (PRRs), including toll-like receptor 4 (TLR4), which subsequently activates downstream signal transduction pathways such as mitogen-activated protein kinases (MAPKs), which leads to the activation of transcriptional factors such as nuclear factor (NF)-κB, interferon regulatory factor (IRF)-3, and activator protein (AP)-1 [7,8,9]. The pathogenesis of inflammation is a complicated process that leads to the production of various molecules and pro-inflammatory products such as nitrite oxide (NO), prostaglandin E_2_ (PGE_2_), tumor necrosis factor α (TNF-α), interleukin-1β (IL-1β), IL-6, and monocyte chemotactic protein-1 (MCP-1) [10,11]. Therefore, these pro-inflammatory mediators are considered important targets for the development of anti-inflammatory agents. For this reason, many studies are currently attempting to develop inhibitors from natural resources to prevent or cure chronic inflammatory conditions that can be used for long periods with minimal side effects [12,13,14].

**Figure 1:**
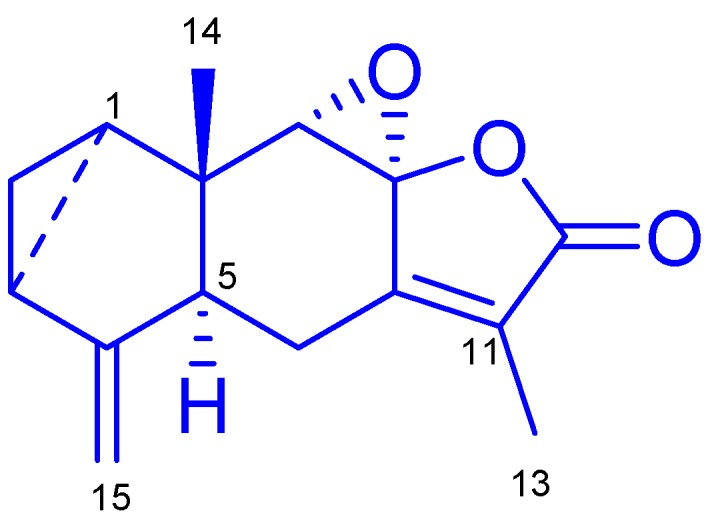
Chemical structure of chloranthalactone B (CTB).

*Sarcandra glabra* (Thunb.) Makino (Chloranthaceae) is an evergreen shrub that is mainly distributed in South of China and Southeast Asia. *S. glabra* is described in the 2010 edition of the Chinese Pharmacopoeia as a traditional medicine for the treatment of inflammation and traumatic injuries [15]. Modern pharmacological research found that the extract of *S. glabra* could protect mice from acute lung injury by inhibiting pro-inflammatory cytokines [16]. Chloranthalactone B (CTB), is a lindenane-type sesquiterpenoid with a molecular weight of 244 and the chemical formula C_15_H_6_O_3_. Its chemical structure is shown in Figure 1. CTB was first isolated from *Chloranthus japonicus* [17], and was found to exhibit inhibitory effects on the generation of superoxide anions by human neutrophils [18]. However, to the best of our knowledge, the mechanisms responsible for the anti-inflammatory effects of CTB are not known. Considering the known pharmaceutical activity of *S. glabra*, we characterized the anti-inflammatory mechanisms of CTB in RAW264.7 macrophages and determined its molecular mechanism of action.

## 2. Results and Discussion

### 2.1. Elucidation of the Chemical Structure of the Isolated Compound

*S. glabra* was extracted in 70% aqueous acetone, and concentrated extract was partitioned into ethyl acetate (EtOAc) and water fractions. Repeated column chromatography of the EtOAc fraction using MCI, SiO_2_, sephadex LH-20, and preparative high performance liquid chromatography (HPLC) yielded compound **1** (Figure 1). The chemical structure of the compound was determined on the basis of spectroscopic analysis, including NMR and MS. Compound **1** was a colorless prism-like crystal and its positive-ion electronic-spray ionization mass spectrometer (ESIMS) produced pseudo-molecular ion peaks [M + Na]^+^ at *m*/*z* 267, consistent with the molecular formula C_15_H_6_O_3_. The ^1^H-NMR spectrum of compound **1** exhibited two methyl groups at δ_H_ 1.90 (3H, s, H-13) and 0.65 (1H, s, H-14), characteristic high-field cyclopropane ring signals at δ_H_ 1.72 (1H, td, *J* = 7.8, 3.6 Hz, H-1), 0.89–0.93 (1H, m, H-2a), 0.83–0.85 (1H, m, H-2b), and 2.00 (1H, m, H-3), and terminal vinyl at 5.03 (1H, br s, H-15a) and 4.70 (1H, br s, H-15b). The above assignments were confirmed by the ^13^C-NMR spectrum, which showed 15 carbon resonance signals including a five-membered α,β-unsaturated lactone ring at δ*_C_* 152.4 (C-7), 88.1 (C-8), 129.3 (C-11), 170.5 (C-12), 9.12 (C-13) (Figures S1 and S2). These results showed that compound **1** was a lindenane sesquiterpene. Compound **1** was identified as chloranthalactone B (CTB) and confirmed by comparison with previous literature [19]. The purity of compound **1** was greater than 95% as determined using HPLC.

### 2.2. The Effects of Chloranthalactone B (CTB) on the Production of Inflammatory Mediators in Lipopolysaccharide (LPS)-Activated RAW 264.7 Cells

**Figure 2:**
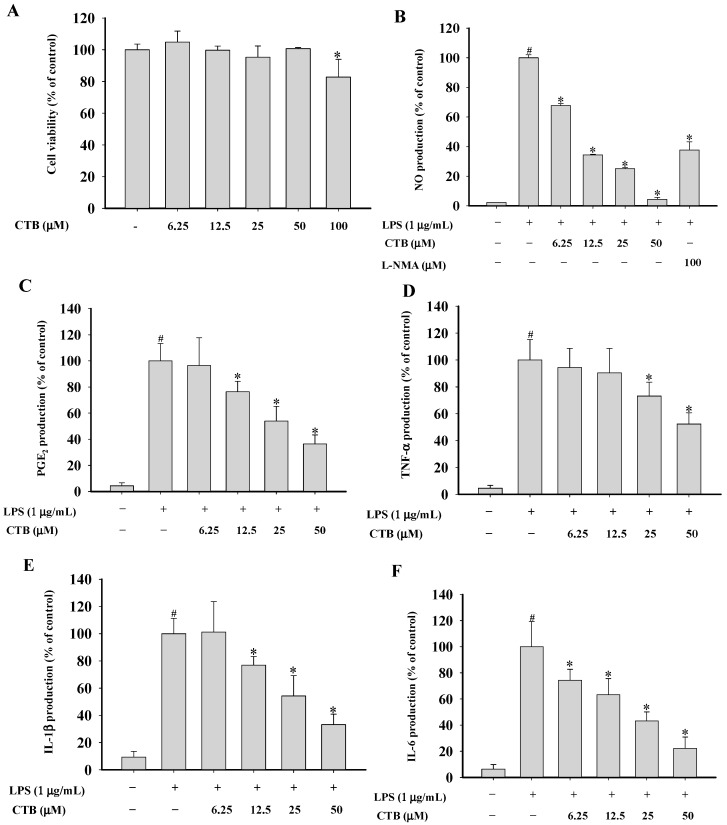
Effects of CTB on cell viability (**A**) and nitric oxide (NO) (**B**); prostaglandin E_2_ (PGE_2_) (**C**); tumor necrosis factor α (TNF-α) (**D**); interleukin-1β (IL-1β) (**E**); and IL-6 (**F**) production in RAW264.7 cells. (**A**) RAW264.7 cells were seeded in 96-well plates and treated with CTB at the indicated concentrations for 24 h. Cell proliferation was estimated by the 1-(4,5-dimethylthiazol-2-yl)-3,5-diphenylformazan (MTT) assay (**A**); RAW264.7 cells were seeded in 96-well plates and pre-treated with CTB for 30 min before the addition of 1 µg/mL LPS for 24 h. Supernatants were collected, and NO levels in culture media were determined using Griess assays (**B**); Levels of PGE_2_, TNF-α, IL-1β, and IL-6 in culture media were quantified using enzyme-linked immunosorbent assay (ELISA) kits (**C**–**F**). Values are the mean ± standard derivation (SD) of triplicate experiments. * *p* < 0.05 compared to LPS treatment alone; ^#^ *p* < 0.05 compared to control group.

Several medications prepared from *S. glabra* are used as anti-tumor or anti-inflammatory drugs in China [20]. Previous investigations of this plant disclosed the presence of bioactive constituents including sesquiterpenes, flavonoids, triterpenoids, coumarins, and phenolic acids [21,22,23,24]. Lindenane and eudesmane-type sesquiterpenoids have been found to be major bioactive components responsible for the anti-inflammatory effects of this herb. A large number of sesquiterpenoids possess anti-inflammatory properties. Cynaropicrin, a sesquiterpene lactone isolated from *Saussurea lappa*, inhibited TNF-α release from LPS-stimulated RAW264.7 and U937 cells [25]. Curcumolide isolated from *Curcuma wenyujin* suppressed LPS-induced nuclear factor (NF)-κB activation and decreased tumor necrosis factor α (TNF-α), interleukin-1β (IL-1β), IL-6, nitrite oxide (NO), and reactive oxygen species (ROS) production [26]. However, there have been few reports on the anti-inflammatory effects of lindenane-type sesquiterpenoids. Our group isolated CTB from the whole plant of *S. glabra*, but the biological activities of this lindenane-type sesquiterpenoid are still relatively unknown. Moreover, no systematic approach has been used to understand the molecular targets and mechanisms underlying the anti-inflammatory effects of CTB. Therefore, this study aimed to characterize the immunopharmacologic mechanisms of CTB action in LPS-stimulated RAW264.7 cells. First, to establish the appropriate concentration ranges of CTB, cells were treated with different concentration of CTB for 24 h followed by 1-(4,5-dimethylthiazol-2-yl)-3,5-diphenylformazan (MTT) assay (Figure 2A). The viability of RAW264.7 cells in each group was 100.00% ± 3.59% (vehicle group), 104.86% ± 6.90% (6.25 µM), 99.68% ± 2.63% (12.5 µM), 95.33% ± 7.07% (25 µM), 100.71% ± 0.70% (50 µM), and 82.76% ± 11.23% (100 µM). No significant differences were observed in cell death rate between 6.25, 12.5, 25, and 50 µM CTB concentrations and the vehicle group. The concentrations used in the subsequent studies were based on these results. Macrophages are the major cellular component and chief effector cells for both specific and non-specific immune responses during inflammation [27]. After macrophages are activated by LPS, a number of different cytokines and other pro-inflammatory molecules are secreted. Accumulating evidence suggests that overproduction of these mediators may be involved in several inflammatory diseases and cancer [28,29]. Thus, inhibition of macrophage activation appears to be an important target for the treatment of inflammatory diseases.

To investigate whether CTB has anti-inflammatory effects in LPS-stimulated RAW264.7 cells, we examined the inhibitory effects of CTB on inflammatory mediator production. As shown in Figure 2B, stimulation with LPS for 24 h resulted in a 42.74-fold increase in NO release macrophages, which was set as the 100% response. Treatment with CTB dramatically inhibited LPS-induced NO production in a dose-dependent manner. NG-methyl-l-arginine (l-NMA), a nonspecific inducible nitric oxide synthase (iNOS) blocker, was used as a positive control to compare the activity of CTB. l-NMA (100 µM) inhibited NO production by 62.35% in LPS-stimulated RAW264.7 cells. Similar activity was obtained with CTB, which reduced NO production by 65.57% at 12.5 µM. Furthermore, we determined the effects of CTB on LPS-induced production of prostaglandin E_2_ (PGE_2_), TNF-α, IL-1β, and IL-6 using an enzyme-linked immunosorbent assay (ELISA) (Figure 2C–F). LPS treatment resulted in significant increases in the production of PGE_2_, TNF-α, IL-1β, and IL-6. Treatment with CTB considerably inhibited the production of pro-inflammatory mediators compared to the LPS-treated control group. These data indicate that CTB significantly inhibits LPS-induced production of the key inflammatory mediators in macrophages without affecting cell viability, suggesting that it is a potential inhibitor of the initial inflammatory response to LPS stimulation.

### 2.3. Effects of CTB on the Expression of Inflammatory Genes and Their Transcriptional Activation

**Figure 3:**
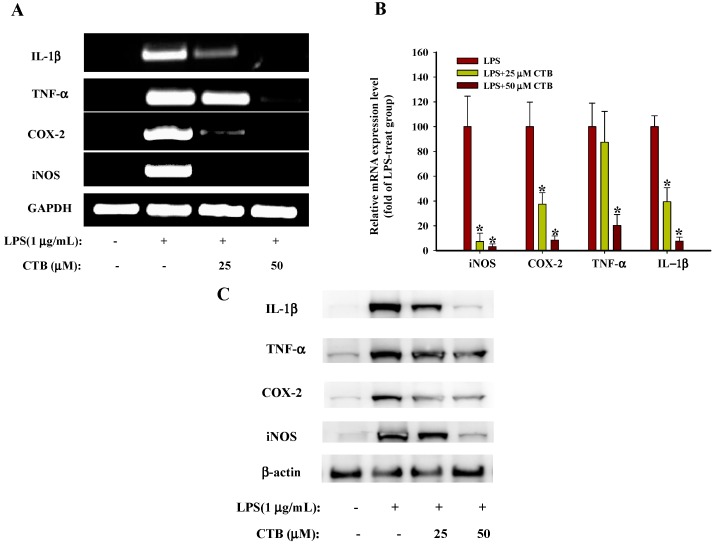
Effects of CTB on inducible nitric oxide synthase (iNOS), cyclooxygenase-2 (COX-2), tumor necrosis factor α (TNF-α), and interleukin-1β (IL-1β) expression in LPS-treated RAW264.7 cells. RAW264.7 cells were pre-treated with different concentrations of CTB (25, 50 µM) for 30 min before treatment with 1 µg/mL LPS for 6 h. iNOS, TNF-α, COX-2, and IL-1β mRNA expression was measured by semi-quantitative PCR (**A**) and real-time PCR (**B**); iNOS, TNF-α, COX-2, and IL-1β protein expression was measured by western blot (**C**). A typical experiment from three independent experiments is shown. * *p* < 0.05 compared to LPS treatment alone.

NO synthesis is regulated by three isoforms of NOS, such as neuronal NOS (nNOS), endothelial NOS (eNOS), and iNOS. iNOS expression is closely associated with pathophysiological conditions such as inflammation. During inflammation, it is overexpressed and an overabundance of NO further increases inflammation [30]. Cyclooxygenase (COX)-2, an inducible isoform of cyclooxygenase, catalyzes PGE_2_ production at the inflammatory site [31]. TNF-α is an inflammatory cytokine synthesized in macrophages that stimulates the production of IL-1β and IL-6. Thus, inflammation is amplified by TNF-α secretion [32]. 6′-*O*-caffeoyldihydrosyringin isolated from *Aster glehni* suppresses LPS-induced iNOS, COX-2, TNF-α, IL-1β and IL-6 expression via NF-κB and AP-1 inactivation in RAW264.7 macrophages [33]. Casticin, isolated from *Vitex rotundifolia* inhibits COX-2 and iNOS expression via suppression of NF-κB and MAPK signaling in LPS-stimulated mouse macrophages [34]. Therefore, blocking NO, PGE_2_, and TNF-α production by inhibiting mRNA expression may be a useful strategy for the treatment of various inflammatory disorders. To investigate whether the inhibitory effects of CTB on inflammatory mediators were associated with the regulation of iNOS, TNF-α, COX-2, and IL-1β expression. RAW264.7 cells were pre-treated with various concentrations of CTB (25 and 50 µM) for 30 min, followed by treatment with LPS for 6 h. Semi-quantitative RT-PCR (Figure 3A) and real-time PCR (Figure 3B) showed that mRNA levels of iNOS, TNF-α, COX-2, and IL-1β were undetectable in RAW264.7 cells without LPS stimulation. The addition of LPS led to a significant increase in iNOS, TNF-α, COX-2, and IL-1β expression, whereas co-treatment with CTB significantly decreased the expression of iNOS, TNF-α, COX-2, and IL-1β compared to the LPS-treated positive control. CTB treatment at 25 µM completely suppressed iNOS mRNA expression, whereas CTB had less of an effect on TNF-α expression. Moreover, CTB downregulates LPS-induced iNOS, TNF-α, COX-2, and IL-1β protein expression levels (Figure 3C), which is consistent with the inhibitory effects of CTB on transcriptional levels. 

**Figure 4:**
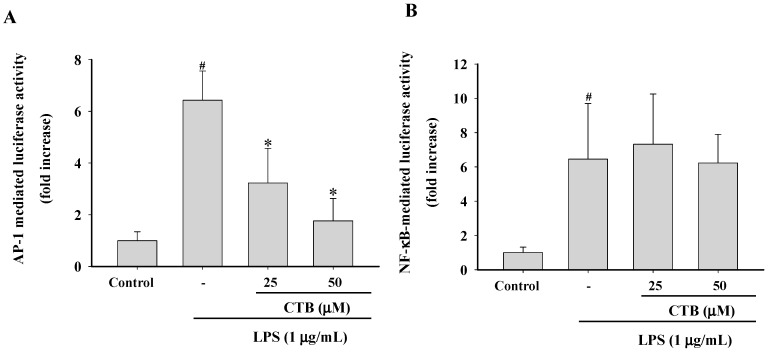
Effects of CTB on transcription factor translocation. RAW264.7 cells co-transfected with plasmids containing AP-1-luc (**A**) or NF-κB (**B**) luciferase constructs were treated with CTB in the presence or absence of LPS. Luciferase activity was determined by luminometry. Values are the mean ± SD of triplicate experiments. * *p* < 0.05 compared to LPS treatment alone; ^#^ *p* < 0.05 compared to control group.

AP-1 and NF-κB are major transcription factors that control inflammatory gene expression and mediate the inflammatory response [35]. Upon activation, cytoplasmic NF-κB and AP-1 translocate to the nucleus, where they mediate the expression of many pro-inflammatory genes [36]. Therefore, we employed a luciferase reporter gene assay using NF-κB-Luc and AP-1-Luc constructs in LPS-stimulated RAW264.7 cells. Cells were transfected with a luciferase reporter construct for AP-1 and NF-κB and produced strong luciferase activity following stimulation by LPS (Figure 4A,B), indicating that LPS activated AP-1- and NF-κB-mediated transcription in RAW264.7 cells. Pretreatment with CTB (25 and 50 µM) resulted in the inhibition of LPS-induced AP-1 activation in a concentration-dependent manner. However, LPS-induced NF-κB luciferase activity was not suppressed by this compound. Other phytochemicals such as ligustilide, costunolide, and saucerneol F also display AP-1-targeted anti-inflammatory activities [37,38,39]. Therefore, these results suggest that AP-1 may be involved in the inhibitory activity of CTB on the expression of inflammatory genes.

### 2.4. Effect of CTB on Upstream Signaling of AP-1

**Figure 5:**
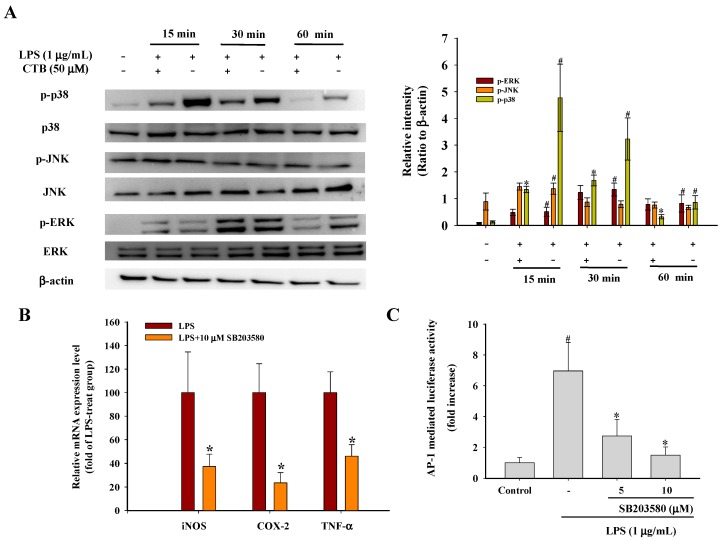

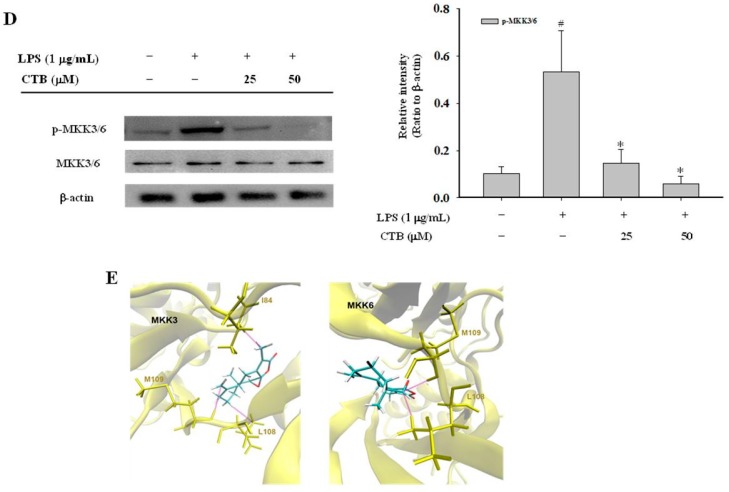
Effects of CTB on the upstream pathway of activator protein (AP)-1. RAW264.7 cells were pre-treated with 50 µM CTB for 30 min and then stimulated with 1 µg/mL LPS for the indicated time. After immunoblotting, the levels of phospho- or total forms of ERK, JNK and p38 were identified based on their antibodies (**A**); RAW264.7 cells were pre-treated with 10 µM p38 MAPK inhibitor (SB203580) for 30 min before treatment with 1 µg/mL LPS for 6 h. mRNA expression of iNOS, COX-2, and TNF-α was measured by real-time PCR (**B**); RAW264.7 cells co-transfected with plasmids containing AP-1-luc luciferase construct were treated with CTB in the presence or absence of LPS (**C**); Values are the mean ± SD of triplicate experiments. * *p* < 0.05 compared to LPS treatment alone; ^#^ *p* < 0.05 compared to control group. RAW264.7 cells were pre-treated with different concentrations of SB203580 for 30 min and then stimulated with 1 µg/mL LPS for 1 h. Cell lysates were immunoblotted with phospho- or total MKK3/6 (**D**); Superposition of the crystal structures of MKK3 and MKK6 with the docking structure of the CTB (**E**). A typical experiment of three independent experiments is shown.

Mitogen-activated protein kinases (MAPKs) are a highly conserved family of protein serine/threonine kinases that include three main signaling cascades: extracellular signal-regulated kinase 1/2 (ERK1/2), p38, and c-Jun N-terminal kinase (JNK) kinase [40]. Several studies have demonstrated that MAPKs are involved in many biological processes, including inflammation, apoptosis, cell growth, and differentiation, and are particularly activated in response to cytokines and stress [41]. In addition, MAPK signaling cascades are activated by LPS through binding of LPS to the TLR4 receptor, which is a critical factor in the signal transduction pathways involved in controlling inflammatory mediators in macrophages [42]. Several lines of evidence have shown that the activation of ERK1/2, p38, and JNK is involved in the regulation of AP-1 activity by increasing transcription [43,44]. To investigate the molecular mechanisms involved in the inhibition of inflammatory gene expression and their transcriptional activation, we examined the effects of CTB on LPS-induced MAPK phosphorylation in RAW264.7 cells using Western blot assays. As shown in Figure 5A, LPS significantly increased the phosphorylation of ERK, JNK, and p38 in RAW264.7 cells within 15 min. This activation was sustained in all of the signaling pathways for at least 1 h. Pre-treatment of cells with 50 μM CTB strongly attenuated the phosphorylation of LPS-induced p38; however, it had no effect on ERK or JNK phosphorylation. Moreover, the amounts of non-phosphorylated ERK1/2, p38, and JNK were not changed by either LPS or CTB pretreatment. Thus, we further confirmed the possible involvement of p38 MAPK in iNOS, COX-2, and TNF-α expression using SB203580 (a specific p38 inhibitor) in LPS-stimulated RAW264.7 cells. We further confirmed relative mRNA expression of iNOS, COX-2, and TNF-α expression by real-time PCR as well as luciferase activity of AP-1 reporter promoter. As expected, SB203580 inhibited LPS-induced iNOS, COX-2, and TNF-α expression in LPS-activated RAW264.7 cells, similar to that observed with CTB treatment (Figure 5B). Moreover, SB203580 decreased the luciferase activity of AP-1 reporter promoter (Figure 5C). This is in line with previous reports that SB203580 suppressed the expression of pro-inflammatory cytokines [45,46]. These results suggest that phosphorylation of p38 MAPK is involved in the inhibitory effects of CTB on LPS-induced inflammatory gene expression. Next, we moved forward to test the activation of phosphorylations of MKK3/6, which are two closely related kinases that phosphorylate p38 at its Thr-Gly-Tyr site [47]. As shown in Figure 5D, the increased phosphorylation of MKK3/6 induced by LPS was suppressed by CTB in a dose-dependent manner, but that this did not affect the total amount of MKK3/6. Furthermore, a molecular modeling study provides the possibility that CTB could inhibit MKK3 and MKK6 by docking the binding site with predicted interaction energies of −4.73 kcal/mol and −5.14 kcal/mol, respectively (Figure 5E). All these observations suggested that CTB could suppress the LPS-mediated AP-1 and p38 MAPK pathways.

## 3. Materials and Methods

### 3.1. Plant Material and Chemicals

The plant materials of *Sarcandra glabra* was collected from Jiujiang region of Jiangxi Province, China and identified by Ce-ming Tan (Jiujiang Institute of Forest Plants, Jiujiang, China). A voucher specimen (accession number CSH20090527) was deposited in Institute of Medicinal Plant Development. LPS (*E. coli* 0111:B4), NG-monomethyl-l-arginine (l-NMA), Griess reagent, dimethyl sulfoxide (DMSO), sodium nitrite (NaNO_2_), and MTT were obtained from Sigma (St. Louis, MO, USA). The kit for RNA isolation and moloney murine leukaemia virus (M-MLV) reverse transcriptase RNasOUT was purchased from Invitrogen (Carlsbad, CA, USA). Primers for *iNOS*, *COX-2*, *IL-1β*, and *TNF-α* were obtained from Generay Biotech (Shanghai, China). The Roswell Park Memorial Institute (RPMI) 1640 medium was obtained from Gibco BRL (Life Technologies, Shanghai, China). Trypsin-EDTA and penicillin–streptomycin solution were acquired from Gibco BRL (Grand Island, NY, USA). Fetal bovine serum (FBS) was acquired from HyClone (Thermo Fisher Scientific, Logan, UT, USA). The phospho-specific ERK (Cat. NO. 8544), JNK (Cat. NO. 4671), MKK3/6 (Cat. NO. 9231), p38 (Cat. NO. 9215), and the total antibodies to ERK (Cat. NO. 4348), JNK (Cat. NO. 9258), MKK3/6 (Cat. NO. 9264), p38 (Cat. NO. 9212), COX-2 (Cat. NO. 12282), TNF-α (Cat. NO. 11948), and IL-1β (Cat. NO. 12426) were obtained from Cell Signaling Technology (Beverly, MA, USA). Antibody to iNOS (Cat. NO. ab178945) was purchased from Abcam (Cambridge, MA, USA). β-actin (Cat. AM1021B) was obtained from Abgent (Suzhou, China). The enhanced chemiluminescence (ECL) Western blotting substrate and Es Taq MasterMix was purchased from ComWin Biotech (Beijing, China). ELISA kits for PGE_2_ (Cat. ab133021), TNF-α (Cat. ab100747), IL-6 (Cat. ab100712), and IL-1β (Cat. ab100704) were purchased from Abcam. Polyvinylidene fluoride (PVDF) membrane was acquired from Bio-Rad (Hercules, CA, USA). All other chemicals were of analytical grade.

### 3.2. General Experimental Procedures

NMR spectra were acquired on a Bruker AV III 600 spectrometer. ESIMS data were recorded on a Thermo Fisher LTQ-Orbitrap XL mass spectrometer. Preparative HPLC was carried out using a Lumtech K1001 analytic LC and a YMC semi-preparative ODS column (10 mm × 250 mm, 5 µm). 

Open-column chromatography was performed with Sephadex LH-20 (Pharmacia Biotech, Uppsala, Sweden) and silica gel (100~200 and 300~400 mesh, Qingdao Marine Chemical Factory, Qingdao, China). Thin-layer chromatography (TLC) was performed on GF_254_ plates (Zhi Fu Huang Wu Pilot Plant of Silica Gel Development, Yantai, China).

### 3.3. Extraction, Fractionation, and Isolation

The air-dried powder of plant materials (5 kg) was extracted with 70% aqueous acetone (30 L) at room temperature for three times. The aqueous residues were combined and evaporated using a vacuum rotary evaporator to produce a crude extract (420 g). The crude extract was suspended in water and then partitioned with ethyl acetate (EtOAc). The EtOAc-soluble fraction (150 g) was chromatographed on an MCI gel column (H_2_O–MeOH, 0%–100%) to afford four fractions (F1–F4). Combined fraction F2 (60 g) and F3 (2.3 g) was subjected to silica gel column chromatography eluted with petroleum ether/EtOAc with a gradient polarity to give eight fractions (Fractions A–H). Fraction E (6.2 g) was separated by Sephadex LH-20 column eluted with MeOH followed by pre-HPLC with MeOH–H_2_O to give CTB (12 mg). 

CTB: Colorless crystal; positive ESI-MS *m*/*z* 267 [M + Na]^+^; ^1^H-NMR (600 MHz, CDCl_3_, δ_H_) 1.72 (1H, td, *J* = 7.8, 3.6 Hz, H-1), 0.89–0.93 (1H, m, H-2a), 0.83–0.85 (1H, m, H-2b), 2.00 (1H, m, H-3), 3.38–3.40 (1H, m, H-5), 2.54 (1H, dd, *J* = 18.0, 12.1 Hz, H-6a), 2.09–2.14 (1H, m, H-6b), 4.18 (1H, s, H-9), 1.90 (3H, s, H-13), 0.65 (3H, s, H-14), 5.03 (1H, br s, H-15a), 4.70 (1H, br s, H-15b); ^13^C-NMR (150 MHz, CDCl_3_, δ_C_) 23.2 (C-1), 17.0 (C-2), 23.2 (C-3), 150.2 (C-4), 50.8 (C-5), 21.5 (C-6), 152.5 (C-7), 88.1 (C-8), 64.6 (C-9), 41.4 (C-10), 129.3 (C-11), 170.5 (C-12), 9.12 (C-13),16.9 (C-14), 106.9 (C-15).

### 3.4. Cell Line and Cell Culture

RAW264.7 macrophages were purchased from American Type Culture Collection (Manassas, VA, USA). Cells were cultured in RPMI 1640 culture medium supplemented with 10% heat-inactivated FBS and 1% penicillin-streptomycin, and incubated at 37 °C in a 5% CO_2_ incubator (HERAcell 150i, Thermo Scientific, Waltham, MA, USA).

### 3.5. Cell Viability Assay

The cytotoxicity of CTB on the RAW264.7 cells determined colorimetrically using MTT assay [48]. Briefly, RAW264.7 cells were seeded at the density of 1 × 10^5^ cells/well in 96-well culture plates and incubated for 24 h, followed by the pre-treatment with increasing concentrations of the CTB (0–50 μM) for 24 h. After that, the medium was removed carefully and cells were treated with media containing 100 μg/mL MTT for 4 h. After incubation, the colored formazan crystals were solubilized with 500 μL of MTT stop solution that contains 10% SDS and 0.01 M hydrochloric acid. The optical densities (OD) were measured using a microplate reader (Infinite M200 Pro spectrophotometer, Tecan, Männedorf, Switzerland) at a wavelength of 550 nm. The optical density of formazan formed in control (vehicle-treated control) cells was considered to be 100% of viability.

### 3.6. Determination of NO, PGE_2_, TNF-α, IL-6, and IL-1β Production

RAW264.7 cells were seeded at the density of 1 × 10^5^ cells/well in 96-well culture plates and incubated for 24 h for adherence. Then, the medium was removed and pretreated with samples for 30 min, followed by stimulated with LPS (1 µg/mL) for an additional 24 h. The production of NO, PGE_2_, TNF-α, IL-6, and IL-1β in the supernatant was quantified using the Griess reagent and ELISA kits as described previously [8].

### 3.7. RNA Extraction and Reverse-Transcription Polymerase Chain Reaction (RT-PCR)

The RAW264.7 cells were plated at a density of 5 × 10^6^ cells/well in 6-well plate overnight. The cells pretreated with CTB for 30 min were incubated with LPS (1 µg/mL) for 6 h. Total RNA was isolated with TRI reagent according to the manufacturer’s instructions. The content of the RNA samples was determined by a UV spectrophotometer (Nanodrop 2000c, Thermo Scientific, Wilmington, DE, USA) and purity was verified by calculation of A260/A280 ratio. An amount of 2 µg total RNA was reverse transcribed to cDNA using M-MLV reverse transcriptase. Quantitative and semi-quantitative PCR were performed as reported previously [49]. The sequences of the primer pairs used in this study are listed in Table 1.

### 3.8. Plasmid Transfection and Luciferase Assay

The RAW264.7 cells were seeded at 5 × 10^5^ cells/mL on 24-well plates and allowed to adhere for 24 h. Cells were transfected with NF-κB-Luc or AP-1-luc reporter plasmid by using the Lipofectamine 3000 (Invitrogen) according to the manufacturer's instructions. At 24 h after transfection, the cells were washed with fresh medium, pretreated with or without samples for 30 min and then stimulated with LPS (1 µg/mL). After the scheduled treatment, each well was washed with cold-PBS and the cells were lysed and the luciferase activities were determined using the Promega luciferase assay system (Promega, Madison, WI, USA).

### 3.9. Western Blot Analysis

The RAW264.7 cells were plated at a density of 5 × 10^6^ cells/well in 6-well plate overnight. The cells pretreated with CTB for 30 min were incubated with LPS (1 µg/mL) for indicated time. Then, cells were collected by centrifugation and washed once with cold PBS. The washed cell pellets were lysed using cell lysis buffer (ComWin Biotech) containing a Roche Complete protease inhibitor cocktail (Roche Diagnostics Ltd., Mannheim, Germany). The cell lysates were centrifuged at 12,000× *g* for 5 min at 4 °C and the protein concentrations of cell lysates were determined using the bicinchoninic acid (BCA) protein assay kit (ComWin Biotech) and bovine serum albumin (BSA) was used as the standard. Twenty-microgram samples of cell lysates were subjected to sodium dodecyl sulfate (SDS)-polyacrylamide gel electrophoresis (PAGE) and transferred to PVDF membranes. Immunoblotting was performed as described previously [50]. The immunoreactive bands were detected by ECL detection system. Densitometric analysis was done using GelQuant image software (Jerusalem, Israel) and calculated by a ratio to a house-keeping control.

### 3.10. Molecular Docking Study of CTB

The structure of CTB was drawn with the aid of Maestro 8.5 version (Schrödinger, LLC, New York, NY, USA) and converted to 3D structure. The crystal structures of MKK3 (PDB ID: 1LEW) and MKK6 (PDB ID: 2BAL) were obtained from RCSB Protein Data Bank. Protein structures were fixed and optimized using Protein Preparation Wizard and the molecular docking studies of the ligand and protein were performed by Glide 5.0 (Schrödinger, NY, USA, 2008) single precision docking mode (SP) with default parameter settings and binding interaction was analyzed. The best fit ligands with the target protein were ranked based on G score.

### 3.11. Data Analysis

All the results were reported as the mean ± standard derivation (SD). The statistical significant differences between experimental and control groups were performed by one way analysis of variance (ANOVA) followed by unpaired Student’s *t*-test. Results were considered significant at *p*-values < 0.05 and are labeled with an asterisk or a hash-mark. Statistical analyses were performed with SPSS version 19.0 (SPSS Inc., Chicago, IL, USA) for Windows 7.

## 4. Conclusions

In conclusion, this study demonstrates that CTB attenuates the production of pro-inflammatory products and modulates the expression of pro-inflammatory mediators in LPS-stimulated RAW264.7 cells. Moreover, our findings suggest that the inhibitory action of CTB is mediated by the inhibition of AP-1 activation and p38 MAPK phosphorylation. Therefore, these results suggest that CTB should be considered a potential treatment for inflammatory diseases. Large quantities of CTB are now being prepared by a semi-preparative HPLC method, and in vivo efficacy testing and investigation of the overall effects of CTB are warranted.

## Figures and Tables

**Table 1:** Primer sequences and conditions for RT-PCR.

Gene Name	GenBank Accession Number	Primer Sequence (5′–3′)
*GAPDH*	NM_001289726	F: CACTCACGGCAAATTCAACGGCA
		R: GACTCCACGACATACTCAGCAC
*iNOS*	NM_001313921	F: CCCTTCCGAAGTTTCTGGCAGCAG
		R: GGCTGTCAGAGCCTCGTGGCTTTGG
*COX-2*	NM_011198	F: CACTACATCCTGACCCACTT
		R: ATGCTCCTGCTTGAGTATGT
*TNF-α*	NM_013693.3	F: TGCCTATGTCTCAGCCTCTTC
		R: GAGGCCATTTGGGAACTTCT
*IL-1β*	NM_008361	F: TGAAGCAGCTATGGCAACTG
		R: AGGTCAAAGGTTTGGAAGGA

F: forward primer; R: reverse primer.

## Supplementary Materials

Supplementary materials can be found at www.mdpi.com/1422-0067/17/11/1938/s1.

## Abbreviations

CTB	Chloranthalactone B
LPS	Lipopolysaccharide
TNF-α	Tumor necrosis factor α
IL-1β	Interleukin-1β
MAPK	Mitogen-activated protein kinase
PRRs	Pattern-recognition receptors
TLR4	Toll-like receptor 4
(NF)-κB	Nuclear factor-κB
(IRF)-3	Interferon regulatory factor-3
(AP)-1	Activator protein-1
NO	Nitric oxide
PGE_2_	Prostaglandin E_2_
MCP-1	Monocyte chemoattractant protein-1
l-NMA	NG-methyl-l-arginine
MTT	1-(4,5-dimethylthiazol-2-yl)-3,5-diphenylformazan
