# Supplementary material for: Anti-Inflammatory Effects of Chloranthalactone B in LPS-Stimulated RAW264.7 Cells
Source: Int J Mol Sci. 2016 Nov 22;17(11):1938. doi: 10.3390/ijms17111938 (PMC5133933; doi:10.3390/ijms17111938)
Supplement: Supplementary file 1 [file ijms-17-01938-s001.pdf]

Supplementary Materials: Anti-Inflammatory Effects of Chloranthalactone B in LPS-Stimulated RAW264.7 Cells

Xueqin Li, Jun Shen, Yunyao Jiang, Ting Shen, Long You, Xiaobo Sun, Xudong Xu, Weicheng Hu, Haifeng Wu and Gongcheng Wang

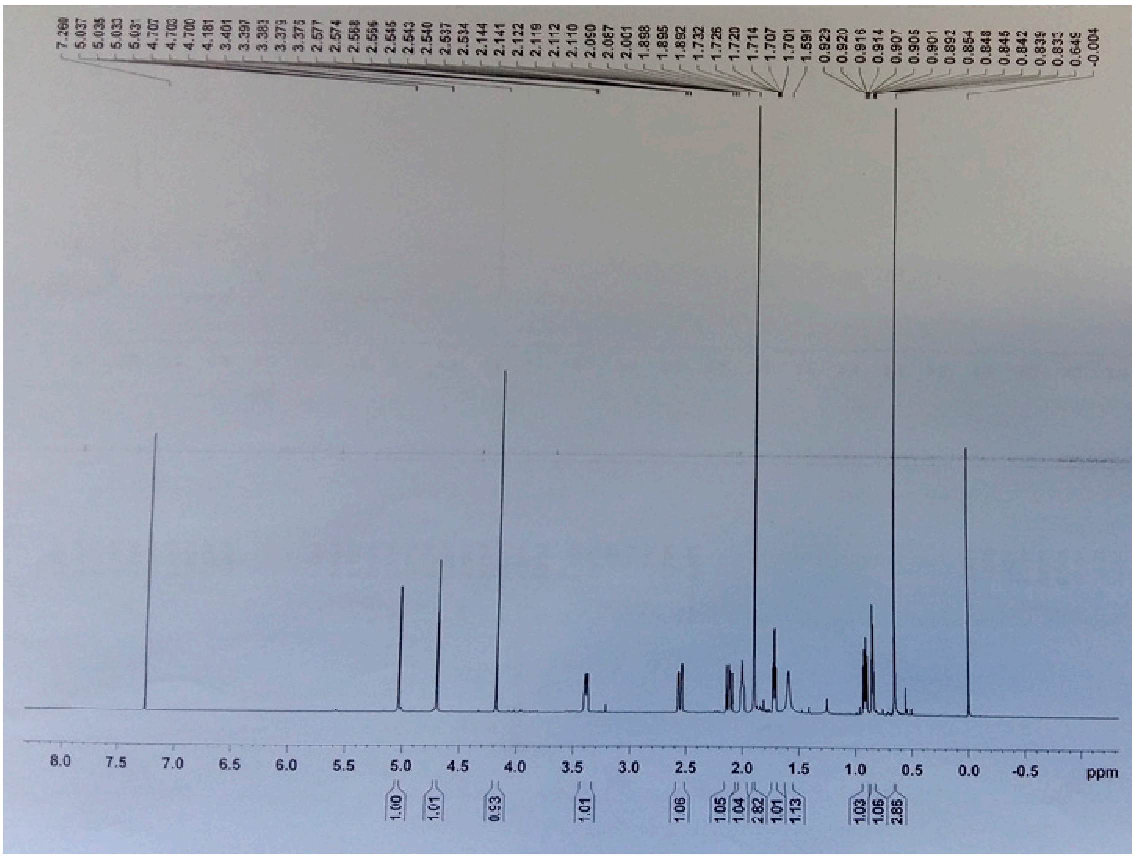

Figure S1. The ^{1}H-NMR (600 MHz, $CDCl_3$) spectrum of chloranthalactone B (CTB).

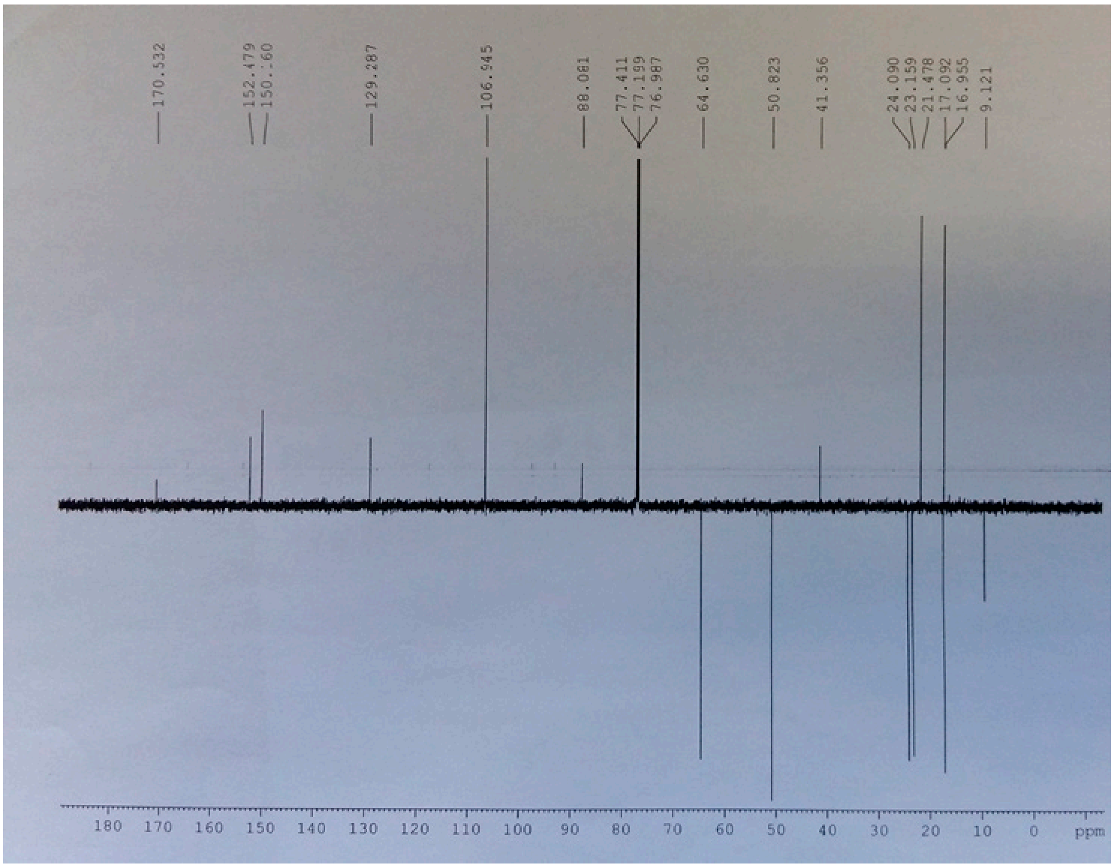

Figure S2. The ^{13}C-NMR (150 MHz, $CDCl_3$) spectrum of CTB.
